# Thymic hyperplasia after chemotherapy for ovarian malignancy: two cases complicated by hyperthyroidism

**DOI:** 10.1093/jscr/rjag048

**Published:** 2026-02-05

**Authors:** Fuyuki Ichikawa, Makoto Koike, Kazuna Matsutani, Asuka Tanaka, Akihiko Yoshimura, Fujihiro Oka, Takeshi Yokoi

**Affiliations:** Department of Obstetrics and Gynecology, Kaizuka City Hospital, 3-10-20 Hori, Kaizuka, Osaka 597-0015, Japan; Department of Obstetrics and Gynecology, Kaizuka City Hospital, 3-10-20 Hori, Kaizuka, Osaka 597-0015, Japan; Department of Obstetrics and Gynecology, Kaizuka City Hospital, 3-10-20 Hori, Kaizuka, Osaka 597-0015, Japan; Department of Obstetrics and Gynecology, Kaizuka City Hospital, 3-10-20 Hori, Kaizuka, Osaka 597-0015, Japan; Department of Obstetrics and Gynecology, Kaizuka City Hospital, 3-10-20 Hori, Kaizuka, Osaka 597-0015, Japan; Department of Obstetrics and Gynecology, Kaizuka City Hospital, 3-10-20 Hori, Kaizuka, Osaka 597-0015, Japan; Department of Obstetrics and Gynecology, Kaizuka City Hospital, 3-10-20 Hori, Kaizuka, Osaka 597-0015, Japan

**Keywords:** thymic hyperplasia, ovarian malignant tumor, chemotherapy, hyperthyroidism, case report

## Abstract

Thymic hyperplasia is a benign condition in which the thymus enlarges in response to various stimuli. It occasionally occurs after chemotherapy for hematologic malignancies or in association with hyperthyroidism, but no previous reports describe cases in which multiple causes coexist. We present two cases of thymic hyperplasia occurring after chemotherapy for ovarian malignancy, both accompanied by concurrent hyperthyroidism. Case 1 was a 29-year-old woman with ovarian dysgerminoma, Stage IC1, and Case 2 was a 46-year-old woman with ovarian high-grade serous carcinoma, Stage IIIB; both patients received surgery and chemotherapy. Twelve and twenty-four months after completion of chemotherapy, computed tomography revealed an anterior mediastinal mass. Additional imaging showed no malignant features. They were diagnosed with thymic hyperplasia. Screening tests later identified hyperthyroidism in both women, and medical therapy was initiated. These cases highlight that thyroid function testing should be considered when thymic hyperplasia is detected in young female patients with malignancy.

## Introduction

Thymic hyperplasia is a benign condition characterized by a transient increase in thymic volume while maintaining normal histological architecture. It is triggered by various physiological or immunological stimuli. Thymic enlargement may occur following chemotherapy for malignant diseases, especially in young patients with hematologic malignancies, and can also occur with disorders such as hyperthyroidism and myasthenia gravis [1–3].

However, reports of thymic hyperplasia occurring after chemotherapy for ovarian malignancy are rare, and no previous reports have described cases in which multiple etiologic factors coexisted in the same patient.

Here, we present two cases of thymic hyperplasia occurring after chemotherapy for ovarian malignancy, both of which were accompanied by concurrent hyperthyroidism.

## Case 1

**Figure 1:**
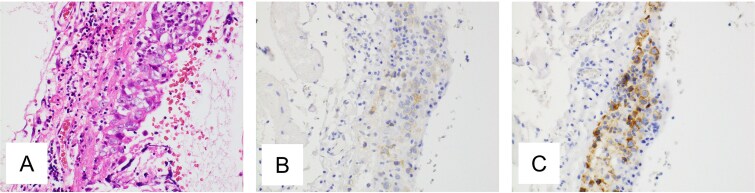
Pathological findings of ovarian tumor for Case 1. (A) Hematoxylin and eosin staining, ×400 show tumor growing in the ovarian cyst. Most of the tumor consists of a mature cystic teratoma, but a focal area demonstrates atypical cell proliferation with surrounding lymphocytic infiltration. (B, C) Immunohistochemical staining, ×400 is positive for c-kit protein and placental alkaline phosphatase.

A 29-year-old woman underwent laparoscopic unilateral ovarian cystectomy for a suspected 5-cm mature cystic teratoma. Histopathological examination revealed dysgerminoma, Stage IC1 (Fig. 1). She declined the recommended additional surgery involving unilateral salpingo-oophorectomy and omentectomy, and instead received adjuvant chemotherapy with bleomycin, cisplatin, and etoposide for a total of three cycles. Post-treatment computed tomography (CT) demonstrated no evidence of disease (NED).

**Figure 2:**
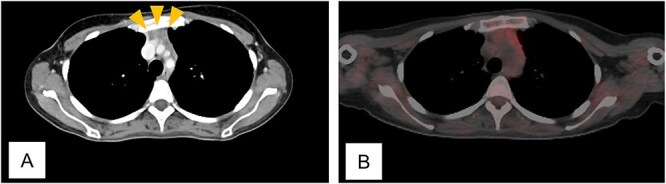
Computed tomogram and fluorodeoxyglucose positron emission tomogram/computed tomogram of Case 1. (A) Chest contrast-enhanced computed tomogram shows a 2 x 1 cm anterior mediastinal mass with increased fat attenuation (arrowhead). (B) No abnormal uptake is seen in the thymic mass on fluorodeoxyglucose positron emission tomogram/computed tomogram.

Twelve months after completion of chemotherapy, follow-up CT revealed an anterior mediastinal mass and enlargement of a left axillary lymph node (Fig. 2A). A biopsy of the axillary node showed no malignant cells. Fluorodeoxyglucose positron emission tomography/CT (FDG-PET/CT) demonstrated no abnormal uptake (Fig. 2B). A diagnosis of thymic hyperplasia was made.

Because she desired future pregnancy but had irregular menstrual cycles, she underwent screening laboratory evaluation. The results showed elevated free T4 and suppressed thyroid-stimulating hormone (TSH), consistent with hyperthyroidism. Further assessment led to a diagnosis of Graves’ disease, for which medical therapy was initiated. She subsequently began fertility treatment. Eighteen months after chemotherapy, she remains without evidence of disease.

## Case 2

**Figure 3:**
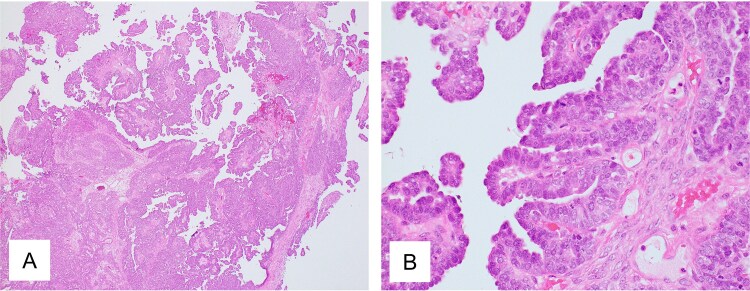
Pathological findings of ovarian tumor for Case 2. (A) Hematoxylin and eosin staining, ×40 and (B) hematoxylin and eosin staining, ×400 show peritoneal dissemination of the ovarian tumor. Morphologically, the tumor shows large papillary structures characteristic of adenocarcinoma, representing a typical appearance of high-grade serous carcinoma.

A 46-year-old woman with ovarian cancer received neoadjuvant chemotherapy with paclitaxel and carboplatin for a total of three cycles. She subsequently underwent total abdominal hysterectomy, bilateral salpingo-oophorectomy, and omentectomy. Histopathological examination revealed high-grade serous carcinoma, stage IIIB (Fig. 3). Homologous recombination deficiency testing was performed and returned positive. After surgery, she received adjuvant chemotherapy with paclitaxel, carboplatin, and bevacizumab for a total of three cycles. Post-treatment CT showed NED. She then received maintenance therapy with olaparib and bevacizumab for 15 months.

**Figure 4:**
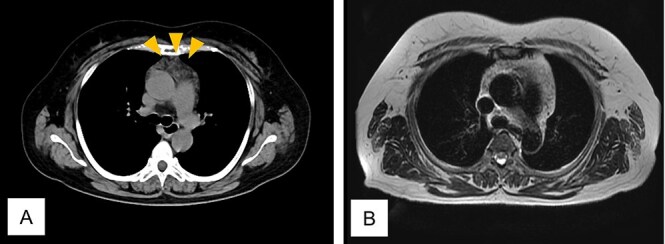
Computed tomogram and magnetic resonance image of Case 2. (A) Chest computed tomogram shows a 4 x 3 cm anterior mediastinal mass with increased fat attenuation (arrowhead). (B) T2-weighted magnetic resonance image of the chest shows heterogeneous fatty signal within the thymus, but no solid component is observed.

Twenty-four months after completing chemotherapy, CT revealed an anterior mediastinal mass (Fig. 4A). Chest magnetic resonance imaging (MRI) demonstrated no malignant features (Fig. 4B). A diagnosis of thymic hyperplasia was made.

She underwent screening tests for underlying conditions, including thyroid function. The results showed elevated free T4 and suppressed TSH, consistent with hyperthyroidism. Further assessment led to a diagnosis of Graves’ disease, for which medical therapy was initiated. Three years after chemotherapy, she remains without evidence of disease.

## Discussion

In this report, two key points are highlighted. First, thymic hyperplasia can rarely develop after chemotherapy for ovarian malignancy. Second, other conditions, including hyperthyroidism, may coexist in patients with thymic hyperplasia.

First, thymic hyperplasia can rarely develop after chemotherapy for ovarian malignancy. Thymic hyperplasia is thought to result from a rebound phenomenon during immune recovery after chemotherapy-induced immunosuppression [4].

To date, only four cases of thymic hyperplasia occurring after chemotherapy for ovarian malignancy have been reported [5–8]. With the addition of the two cases presented here, the characteristics are summarized in Table 1. Notably, most patients were young and had germ cell tumors. Thymic hyperplasia typically developed within one year after chemotherapy. All cases were asymptomatic, and anterior mediastinal masses were incidentally detected on CT. Historically, diagnosis was made by thymectomy, but in recent years, imaging studies have been sufficient for diagnosis. All patients had NED from their ovarian tumors.

**Table 1:** Case reports of thymic hyperplasia occurring after chemotherapy for ovarian malignancy

Case	Author (year)	Age	Diagnosis of ovarian tumors	Stage (FIGO 2014)	Operation	Chemotherapy	Symptoms of thymic hyperplasia	Trigger of diagnosis	Time of diagnosis	Other image diagnosis (Modality)	Histological diagnosis (Procedure)	Other causative diseases	Outcome of ovarian tumors
1	Carmosino (1985)	23	Endodermal sinus tumor	IIB-IIIC	TAH + BSO + OM	BLM + VBL + CDDP	Asymptomatic	Mediastinal mass on XP and CT	8 months after chemotherapy	None	Thymic hyperplasia (Thymectomy)	None	NED
2	Achir (2010)	31	Endodermal sinus tumor	IIB-IIIC	TAH + OM	BLM + VP-16 + CDDP	Asymptomatic	Mediastinal mass on CT	6 months after chemotherapy	None	Thymic hyperplasia (Thymectomy)	None	NED
3	Guida (2013)	16	Dysgerminoma	IIIC	USO	BLM + VP-16 + CDDP	Asymptomatic	Mediastinal mass on CT	2 months after chemotherapy	No abnormal glucose uptake (FDG-PET/CT)	None	None	NED
4	Khan (2018)	24	Mixed germ cell tumor (recurrence)	IVB	USO, PALD+PLD + PH + LL	PTX + CDDP+IFM, VP-16 + CBDCA	Asymptomatic	Mediastinal mass on CT	Immediately after chemotherapy	None	Normal thymus tissue (Biopsy)	None	NED
5	Present case 1	29	Dysgerminoma	IC1	UC	BLM + VP-16 + CDDP	Asymptomatic	Mediastinal mass on CT	12 months after chemotherapy	No abnormal glucose uptake (FDG-PET/CT)	None	Hyperthyroidism	NED
6	Present case 2	46	High-grade serous carcinoma	IIIB	TAH + BSO + OM	PTX + CBDCA+BV, OLA + BV	Asymptomatic	Mediastinal mass on CT	24 months after chemotherapy	No malignant findings (MRI)	None	Hyperthyroidism	NED

BLM, Bleomycin; BSO, Bilateral salpingo-oophorectomy; BV, Bevacizumab; CBDCA, Carboplatin; CDDP, Cisplatin; CT, Computed tomography; FDG-PET/CT, Fluorodeoxyglucose positron emission tomography/CT; IFM, Ifosfamide; LL, Lung lobectomy; MRI, Magnetic resonance imaging; NED, No evidence of disease; OLA, Olaparib; OM, Omentectomy; PALD, Para-aortic lymphadenectomy; PH, Partial hepatectomy; PLD, Pelvic lymphadenectomy; PTX, Paclitaxel; TAH, Total abdominal hysterectomy; UC, Unilateral cystectomy; USO, Unilateral salpingo-oophorectomy; VBL, Vinblastine; VP-16, Etoposide; XP, X-ray photograph.

Thymic hyperplasia must be distinguished from malignant conditions such as thymic metastasis; however, thymectomy is invasive, and CT may be insufficient for differentiating benign from malignant conditions. Imaging modalities such as MRI and FDG-PET/CT have been reported to be useful for noninvasive diagnosis [9–11]. In addition, in patients with lung cancer or malignant lymphoma who developed thymic hyperplasia, a favorable prognosis was suggested [12, 13], and our findings indicate that a similar pattern may apply to ovarian malignancy.

Second, other conditions, including hyperthyroidism, may coexist in patients with thymic hyperplasia. The mechanism is not fully understood, but excessive thyroid hormone and activation of thymic TSH receptors are thought to contribute to thymic enlargement in hyperthyroidism [14]. In previously reported cases of chemotherapy-associated thymic hyperplasia, no patient was reported to have a concurrent condition such as hyperthyroidism.

Hyperthyroidism occurs more frequently in young women and can cause menstrual irregularities and anovulation, potentially leading to infertility. In young women desiring pregnancy, such as the patient in Case 1, the presence of thymic hyperplasia should prompt consideration of concurrent hyperthyroidism, and appropriate screening tests should be performed.

In conclusion, thymic hyperplasia can rarely occur after chemotherapy for ovarian malignancy. In some cases, other conditions that can contribute to thymic enlargement, such as hyperthyroidism, may be present concurrently. When thymic hyperplasia is detected in young female patients with malignancy, screening for thyroid function should be considered.
